# The Usefulness of Two CXCL13 Assays on Cerebrospinal Fluid for the Diagnosis of Lyme Neuroborreliosis: a Retrospective Study in a Routine Clinical Setting

**DOI:** 10.1128/JCM.00255-21

**Published:** 2021-08-18

**Authors:** Tamara van Gorkom, Gijs H. J. van Arkel, Michiel Heron, Willem Voet, Steven F. T. Thijsen, Kristin Kremer

**Affiliations:** a Department of Medical Microbiology and Immunology, Diakonessenhuis Hospital, Utrecht, The Netherlands; b Department of Neurology, Diakonessenhuis Hospital, Utrecht, The Netherlands; c Centre for Infectious Diseases Research, Diagnostics and Laboratory Surveillance, Centre for Infectious Disease Control, National Institute for Public Health and the Environment (RIVM), Bilthoven, The Netherlands; University of Tennessee at Knoxville

**Keywords:** *Borrelia*, Lyme neuroborreliosis, cerebrospinal fluid, CXCL13, Reibergram, blood-CSF barrier functionality, intrathecal antibody synthesis

## Abstract

Recent studies have shown elevated levels of the B-cell chemokine (C-X-C motif) ligand 13 (CXCL13) in the cerebrospinal fluid (CSF) of patients with early Lyme neuroborreliosis (LNB). In this retrospective study, we evaluated the diagnostic performance of the Quantikine CXCL13 enzyme-linked immunosorbent assay (ELISA) (R&D Systems, Inc., MN, USA) and the *recom*Bead CXCL13 assay (Mikrogen, Neuried, Germany) for the detection of CXCL13 in CSF. All consecutive patients from whom a CSF and a serum sample had been collected between August 2013 and June 2016 were eligible for inclusion. Patients suspected of LNB were classified as definite, possible, or non-LNB according to the guidelines of the European Federation of Neurological Societies (EFNS). Due to the limited number of LNB patients in the predefined study period, additional LNB patients were included from outside this period. In total, 156 patients (150 consecutive patients and 6 additional LNB patients) were included. Seven (4.5%) were classified as definite, eight (5.1%) as possible, and 141 (90.4%) as non-LNB patients. Receiver operating characteristic (ROC) curve analysis comparing definite-LNB patients with non-LNB patients showed a cutoff value of 85.9 pg/ml for the Quantikine CXCL13 ELISA and 252.2 pg/ml for the *recom*Bead CXCL13 assay. The corresponding sensitivity was 100% (95% confidence interval [CI], 100% to 100%) for both, and the corresponding specificities were 98.6% (95% CI, 96.5% to 100%) for the CXCL13 ELISA and 97.2% (95% CI, 93.6% to 100%) for the *recom*Bead CXCL13 assay. This study showed that CXCL13 in CSF can be of additional value for the diagnosis of LNB.

## INTRODUCTION

Lyme borreliosis (LB) is a tick-borne disease caused by spirochetes of the Borrelia burgdorferi *sensu lato* group. The most frequent manifestation of LB is a local skin rash referred to as erythema migrans (1, 2). When unnoticed, the *Borrelia* bacterium can spread throughout the body and infect organs such as nerves, joints, or heart. Nervous system involvement, also known as Lyme neuroborreliosis (LNB), occurs in about 2 to 13% of LB cases (3–6). In the Netherlands, the incidence rate of LNB was 2.6 (95% confidence interval [CI], 2.4 to 2.8) per 100,000 inhabitants in 2010, as calculated from a nationwide survey conducted among physicians (4). Patients with clinical symptoms suggestive of LNB often present with manifestations involving the peripheral and/or cranial nerves, such as radiculopathy and/or cranial neuropathy. The diagnosis of LNB should be supported by laboratory analysis. Detection of the *Borrelia* bacterium by PCR or culture is not very sensitive (7–9). Instead, the guidelines set by the European Federation of Neurological Societies (EFNS) recommend the detection of intrathecally produced *Borrelia*-specific antibodies together with the presence of pleocytosis (≥5 leucocytes/μl) in the cerebrospinal fluid (CSF) (10). The interpretation of laboratory tests used for the detection of intrathecally produced *Borrelia*-specific antibodies, however, can be complicated for various reasons. First, the levels of intrathecally produced *Borrelia*-specific antibodies may be too low for detection in the early stages of the disease (<6 weeks) (11, 12). Second, intrathecally produced *Borrelia*-specific antibodies may persist after adequate antibiotic treatment (11, 13). In case of nonspecific clinical symptoms and pleocytosis, the decision to treat often must be taken before the laboratory results on the intrathecal synthesis of *Borrelia*-specific antibodies become available. This underlines the need for new diagnostic tools for LNB, based on markers with high sensitivity and specificity, especially because early antibiotic treatment has proven to be effective (14, 15).

In 2005, elevated levels of the B-cell chemokine (C-X-C motif) ligand 13 (CXCL13) in CSF samples of LNB patients were detected by using a protein expression array (16) and an enzyme-linked immunosorbent assay (ELISA) (17). CXCL13 was shown to be involved in the migration of B-lymphocytes to the CSF (18, 19), and in some cases, elevated levels of CXCL13 preceded the synthesis of *Borrelia*-specific antibodies in the CSF (20, 21). Elevated CSF CXCL13 levels have also been found in the absence of pleocytosis (22, 23). Since 2005, various studies have confirmed that the detection of CXCL13 in CSF is a useful marker for the diagnosis of early LNB, irrespective of the causative *Borrelia* species (24), even though elevated CSF CXCL13 levels have also been found in patients with other bacterial (14, 24–29) and viral (21, 25–28, 30–32, 54) central nervous system (CNS) infections, autoimmune diseases (19, 28, 33), and white blood cell line malignancies (26, 34). As CSF CXCL13 levels decline rapidly after successful antibiotic therapy for LNB, this marker can be used to distinguish an active LNB infection from a previous, cleared infection (16, 20, 25, 28).

Several commercial assays for the detection of CXCL13 are available, and various studies have been performed to investigate the usefulness of CXCL13 in CSF as a marker for the diagnosis of LNB. One of the assays studied the most is the human B-lymphocyte chemoattractant/B cell-attracting chemokine 1 (BLC/BCA-1) immunoassay (Quantikine CXCL13 ELISA; R&D Systems, Inc., Minneapolis, MN, USA). The instruction manual for this assay, however, does not mention the use of CSF for the detection of CXCL13 and consequently lacks a cutoff value for CXCL13 in the CSF. Various studies have shown the potential of the detection of CXCL13 in the CSF for the diagnosis of LNB; however, a broad range of cutoff values was found, ranging from 18 pg/ml to 1,229 pg/ml (14, 20, 21, 23, 25, 26, 28, 35, 36). The diagnostic performance of a test largely depends on the study design (e.g., prospective versus retrospective and/or case-control versus cross-sectional) (37, 38). For most studies, the study population did not match a routine clinical setting, and this is a drawback of many LB test evaluation studies (37). Therefore, the aim of our study was to assess the diagnostic potential of two CXCL13 assays on CSF from all consecutive patients that visited our hospital in a defined time frame and from whom a blood sample was taken as well. We chose an established CXCL13 assay (i.e., the Quantikine CXCL13 ELISA [R&D systems, Inc.]) to evaluate the performance of the *recom*Bead CXCL13 assay (Mikrogen GmbH, Neuried, Germany), which makes use of the innovative Luminex xMAP technology.

## MATERIALS AND METHODS

### Study population.

**FIG 1:**
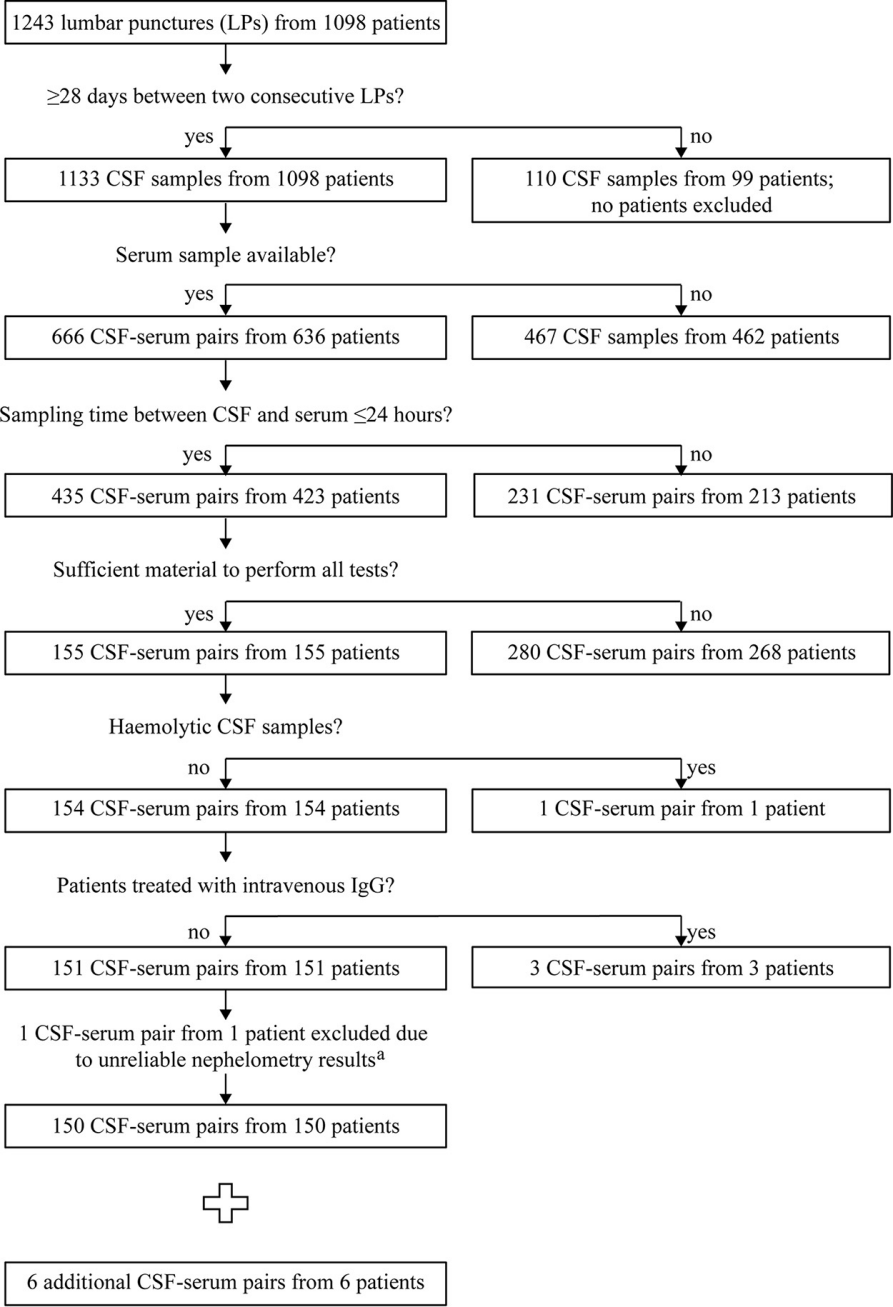
Flow chart of the inclusion of the 156 patients. From all 1,098 consecutive patients with a lumbar puncture in the period between August 2013 and June 2016, 155 (14.1%) had sufficient amounts of CSF and serum that were drawn from the patient within 24 h. Five (3.2%) of the 155 patients were excluded for various reasons. CSF-serum pairs from six additional LNB patients were collected between February 2011 and July 2013 (*n* = 1) or between July 2016 and November 2017 (*n* = 5). ^a^Initially, testing had started with 151 CSF-serum pairs; however, one CSF-serum pair was excluded after the nephelometry results became available. The albumin concentration in the CSF of this CSF-serum pair far exceeded the total protein concentration in CSF, which was measured at the time of diagnosis, 3 months earlier, implying a sample mix-up.

Retrospectively, and irrespective of their clinical suspicion, all consecutive patients from whom a CSF and a blood sample (drawn within 24 h of the lumbar puncture [LP]) had been sent to the microbiology laboratory of the Diakonessenhuis Hospital, Utrecht, the Netherlands, between August 2013 and June 2016 were selected. The patients were divided into three groups based on the criteria for LNB as defined by the EFNS (10). These criteria are (i) the presence of neurological symptoms suggestive of LNB in the absence of other possible causes, (ii) CSF pleocytosis (≥5 leukocytes/μl), and (iii) intrathecal synthesis of *Borrelia*-specific antibodies. If all criteria were met, then the patient was classified as a definite-LNB patient; if two criteria were met, then the patient was classified as a possible-LNB patient. In all other cases, the patient was classified as a non-LNB patient. As the samples that were collected within the framework of this study were also used for another study that evaluated various commercial LNB assays (T. van Gorkom, W. Voet, G. H. J. van Arkel, M. Heron, S. F. T. Thijsen, and K. Kremer, unpublished data), the CSF and serum sample pairs were only included if at least 1,250 μl of CSF and 110 μl of serum were available (Fig. 1). Patients with hemolytic CSF or who were treated intravenously with IgG were excluded from the study, as both could lead to erroneous results (39, 40). For all study participants, information about symptoms, symptom duration prior to the LP, and antibiotic treatment for LNB was assessed through consultation of the electronic patient files. No systematic survey was conducted on the use of antibiotic treatment in the period preceding the LP.

Due to the relatively low number of LNB patients with sufficient material in the predefined study period, additional LNB patients (definite or possible LNB) were chosen from outside the study period (from February 2011 to July 2013 and from July 2016 to November 2017). The LNB patients who were eligible had taken part in two other studies of our research group (41, 42), and consequently, both CSF and serum from the time of diagnosis had been stored. For these additional LNB patients, the same inclusion criteria applied as for all consecutive patients.

All samples used in this study were anonymized. According to the rules of our hospital, our study was exempt from approval of the local Ethics Committee because the main goal was to validate a new assay for the diagnosis of LNB for which leftover material could be used. We did, however, obtain approval from the hospital board to conduct this study.

### Detection of *Borrelia*-specific antibodies.

The detection of *Borrelia*-specific total immunoglobulin (IgM and IgG) in serum was done by using the C6 enzyme-linked immunosorbent assay (ELISA) (Immunetics, Boston, MA, USA), and equivocal and positive C6 ELISA results were confirmed by using the *recom*Line IgM and IgG immunoblot test (Mikrogen Diagnostik GmbH) as described previously (42). The detection of intrathecally produced *Borrelia*-specific IgM and IgG was done by using the second-generation IDEIA LNB assay (Oxoid, Basingstoke, United Kingdom) as described previously (42). To determine the intrathecal synthesis of *Borrelia*-specific IgM (or IgG), CSF and serum of a CSF-serum pair were tested simultaneously in the same run. The IDEIA LNB assay is based on the capture ELISA principle and determines the fraction of *Borrelia*-specific IgM (or IgG) as part of the total amount of IgM (or IgG) in CSF and serum. If the fraction of *Borrelia*-specific IgM (or IgG) in the CSF exceeds the fraction in serum, then intrathecal synthesis of *Borrelia*-specific IgM (or IgG) is proven. Consequently, a correction for a dysfunctional blood-CSF barrier was not needed.

### *Borrelia* species PCR on CSF.

An in-house real-time *Borrelia* species PCR was used, for which the 23S rRNA gene was used as a target. A total of 190 μl of the CSF sample was processed together with an internal control consisting of *Synechococcus* DNA (Dutch Institute of Ecology/Royal National Academy of Sciences, Yrseke, the Netherlands) by using the MagnaPure LC total-nucleic acid, high-performance isolation kit (Roche Diagnostics, Almere, the Netherlands) on a MagnaPure LC instrument (Roche Diagnostics). The amount of internal control used had to result in a threshold cycle value in the diagnostic range, and 10 μl was sufficient, which was established empirically. The isolated DNA was eluted in 100 μl of elution buffer (Roche Diagnostics). The real-time PCR contained 12.5 μl TaqMan universal PCR mastermix (ThermoFisher Scientific, Bleiswijk, the Netherlands), 10 μl of eluted DNA, and 2.5 μl of a primer and probe mix. The primer and probe mix consisted of a 300 nM concentration of the forward primer (5′-GGGCGATTTAGTTAGATGTGGTAGA-3′), a 900 nM concentration of the reverse primer (5′-CAAGCTTCAGCCTGGCCATA-3′), and a 200 nM concentration of the probe (5′-6-carboxyfluorescein [FAM]-AASCCGAGTGATCTAT-3′ minor groove binder [MGB] nonfluorescent quencher). The *Borrelia* species PCR was performed in a 96-well plate (ThermoFisher Scientific) on an ABI 7500 system (ThermoFisher Scientific). The program consisted of 1 cycle of 2 min at 50°C, 1 cycle of 15 min at 95°C, and 45 cycles that consisted of 15 s at 95°C followed by 1 min at 60°C. A positive control (DNA of B. burgdorferi; Vircell Microbiologists, Granada, Spain) and a negative control (elution buffer; Roche Diagnostics) were included in each experiment to control for amplification and contamination.

### Detection of CXCL13 in CSF.

CSF samples of all patients were tested by using two CXCL13 assays according to the manufacturer’s instructions. The first assay was the Quantikine CXCL13 ELISA (R&D Systems, Inc.), hereinafter referred to as the CXCL13 ELISA, which was performed on a Dynex DS2 automated ELISA instrument (Dynex Technologies, Chantilly, VA, USA) and analyzed with the DS-Matrix software (Dynex Technologies). The manufacturer of this CXCL13 ELISA recommends the assay for use in the quantitative measurement of CXCL13 in cell culture supernatants, serum, plasma, and saliva but does not mention its use for CSF (43). The final CSF CXCL13 concentrations (in pg/ml) were calculated by using a standard curve. The standard curve was based on seven standards with concentrations ranging from 7.81 pg/ml to 500 pg/ml. These standards were included in each run, and after the optical densities (ODs) were measured, a curve was constructed by plotting the mean OD value from each standard on the *y* axis and the concentration on the *x* axis as a log/log graph in the Dynex DS2 automated ELISA instrument (Dynex Technologies). Subsequently, the OD values from the CSF samples were interpreted by using this standard curve. Concentrations below the lower detection limit of the standard curve (<7.81 pg/ml) were assigned a value of 7.81 pg/ml, and concentrations above the upper detection limit of the standard curve (>500 pg/ml) were assigned a value of 500 pg/ml.

The second assay was the Luminex-based *recom*Bead CXCL13 assay (Mikrogen Diagnostik GmbH). CXCL13 concentrations (in pg/ml) were measured by using the Luminex xMAP technology. Bead analysis was done on a Bio-Plex 200 instrument (Bio-Rad Laboratories, Hercules, CA, USA) by using the Bio-Plex Manager software, version 6.1 (Bio-Rad Laboratories). The final CXCL13 concentration of a CSF sample was calculated by using a calibrator, together with the batch-dependent 4-parameter logistic (4-PL) coordinates of a standard curve by using the *recom*Quant evaluation software version 4.9.5 (Mikrogen Diagnostik GmbH). The measurement range is located between 9.00 and 1,000 pg/ml. CXCL13 concentrations of less than 9.00 pg/ml were assigned a value of 9.00 pg/ml, and CXCL13 concentrations of more than 1,000 pg/ml were assigned a value of 1,000 pg/ml. According to the instruction manual, CSF CXCL13 concentrations of less than 190 pg/ml are normal, between 190 and 300 pg/ml are borderline, and more than 300 pg/ml are elevated, for which an active LNB infection is suspected if symptoms match (40).

### Blood-CSF barrier functionality, intrathecal total-antibody synthesis, and construction of Reibergrams.

Blood-CSF barrier functionality and intrathecal total-antibody synthesis were assessed by the construction of Reibergrams (44). Therefore, CSF and serum concentrations of albumin, total IgM, and total IgG were determined by nephelometry using the BN ProSpec system (Siemens Healthcare Diagnostics Products GmbH, Marburg, Germany) following the instructions of the manufacturer. By using the website www.albaum.it (45), these concentrations were used to calculate the CSF/serum quotients for albumin (Q Alb), IgM (Q IgM), and IgG (Q IgG), as well as the intrathecal fractions of IgM and IgG. Subsequently, the Q IgM and Q IgG of all patients were plotted relative to the Q Alb. In both the IgM and the IgG Reibergram, the reference ranges for the blood-derived fractions of total IgM and total IgG were shown by nonlinear hyperbolic curves (mean reference line ± 3 standard deviations). These lines best reflect the biological laws of diffusion from the blood as has been described by Reiber (46). In addition, four lines that are located above the upper reference line show 20%, 40%, 60%, and 80% increases of the measured total-antibody concentration in the CSF and thus reflect the fraction of intrathecally produced total antibodies (44). A vertical line can be added to show the age-dependent reference line for Q Alb. In this study, only the mean age-dependent reference line for Q Alb is reported.

Based on the position of Q IgM and Q IgG in the Reibergram, every patient was classified in one of five groups corresponding to the five areas of the Reibergram (44), as follows: area 1, normal CSF findings (total-antibody quotient below the upper reference line for blood-derived total antibodies and left of the age-dependent reference line for Q Alb); area 2, dysfunctional blood-CSF barrier (total-antibody quotient below the upper reference line for blood-derived total antibodies and right of the age-dependent reference line for Q Alb); area 3, dysfunctional blood-CSF barrier and intrathecal total-antibody synthesis (total-antibody quotient above the upper reference line for blood-derived total antibodies and right of the age-dependent reference line for Q Alb); area 4, normal blood-CSF barrier and intrathecal total-antibody synthesis (total-antibody quotient above the upper reference line for blood-derived total antibodies and left of the age-dependent reference line for Q Alb); and area 5, indicative of a methodological error (e.g., unpaired CSF/serum samples, measurement in antigen excess range, etc.). The overall Reibergram classification was based on the combined IgM and IgG results. There was no proof of intrathecal total-antibody synthesis if both the intrathecal total-IgM and the intrathecal total-IgG fraction were equal to or less than 10%. If either one or both intrathecal fractions were larger than 10%, then intrathecal total-antibody synthesis was proven (44).

### Data handling and statistical analysis.

For all statistical analyses, Rstudio (version 1.3.959, 2009-2020) was used (47). The data were analyzed by performing two-group comparisons, for which the coin package was used (48). Nominal data were analyzed by using either the exact Pearson’s chi-squared test or the approximate Monte Carlo resampling (10^6^) Pearson’s chi-squared test, and quantitative data were analyzed by using the exact Wilcoxon-Mann-Whitney test. Results are shown as the (geometric) mean value with the 95% CI or as the median value with the range. For construction of the dot plots, GraphPad Prism (version 8.4.1; GraphPad Software, San Diego, CA, USA) was used.

To assess the diagnostic performance of both CXCL13 assays for the diagnosis of LNB, a receiver operating characteristic (ROC) curve was constructed for each assay separately and used to calculate the area under the curve (AUC) by using the pROC package (49). Therefore, the CXCL13 concentrations in CSF samples of cases (i.e., definite-LNB patients) were compared with those of controls (i.e., non-LNB patients). Subsequently, the optimal cutoff values were calculated by using the point on the ROC curve for which the distance to the upper left corner, where both sensitivity and specificity are 100%, was shortest, and was determined by the square root of [(1 − sensitivity)^2^ + (1 − specificity)^2^]. CSF CXCL13 levels above the optimal cutoff value in each assay were classified as positive. The sensitivity, specificity, positive predictive value (PPV), and negative predictive value (NPV) with 95% CIs for each assay were calculated by using the optimal cutoff value. The performances of both assays were compared by comparison of the ROC curves using the clinfun package and 10^6^ permutations (50).

Raw *P* values of <0.050 were statistically significant; however, they were interpreted after correction for the multiple statistical analyses in this study, for which the Benjamini-Hochberg procedure (BH) was applied (51). The false discovery rate (FDR) was set at the level of 5.0%, and less than one false-positive result was allowed in our list of rejections.

## RESULTS

### Study population.

A flow chart of the study population is shown in Fig. 1. The final study population comprised 156 patients, of whom 150 (96.2%) consisted of all consecutively collected patients who had had an LP in our hospital between August 2013 and June 2016 and from whom sufficient amounts of CSF and serum were collected. These 150 consecutive patients did not significantly differ regarding age and gender from the 1,098 consecutive patients for whom at least one LP was performed in the predefined study period (data not shown). Six additional LNB patients, who were part of two other studies from our research group (41, 42), were selected from outside this period, to increase the number of LNB patients. In total, 78 men and 78 women were included in the study. The mean age of the patients was 52.5 years (95% CI, 49.9 to 55.1), the geometric mean CSF cell count was 1.6 cells/μl (95% CI, 1.1 to 2.3 cells/μl), and the geometric mean symptom duration was 60.3 days (95% CI, 43.0 to 84.0 days). The basic characteristics for each study group are shown in Table 1. As expected, considering the definitions of definite-LNB and possible-LNB cases (10), LNB patients, more often than non-LNB patients, had pleocytosis and *Borrelia*-specific antibodies in their blood. Definite-LNB patients had intrathecally produced *Borrelia*-specific antibodies more often than possible-LNB patients and non-LNB patients (Table 1).

**TABLE 1:** Demographic and clinical characteristics among all 156 patients included in this study

Characteristic^*a*^	Value for indicated patient group^*b*^				Raw *P* value for BH comparison^*e*^		
	All patients (*n* = 156)	dLNB (*n* = 7)^*c*^	pLNB (*n* = 8)^*d*^	Non-LNB (*n* = 141)	1 vs 2	1 vs 3	2 vs 3
Gender [no. of males (%)]	78 (50.0)	6 (85.7)	4 (50.0)	68 (48.2)	0.282	0.116	1.000
Age [mean (95% CI)]	52.5 (49.9–55.1)	68.8 (61.0–76.2)	52.1 (37.9–66.4)	51.8 (49.0–54.5)	0.094	0.007^*f*^	0.776
Pleocytosis							
≥5 Leucocytes/μl [no. (%)]	36 (23.1)	7 (100)	7 (87.5)	22 (15.6)	1.000	<0.001^*f*^	<0.001^*f*^
CSF cell count/μl [geometric mean (95% CI)]	1.6 (1.1–2.3)	67.4 (27.4–164)	24.5 (8.9–66.7)	1.1 (0.8–1.6)	0.179	<0.001^*f*^	<0.001^*f*^
Positive *Borrelia* species PCR on CSF	2 (1.3)	2 (28.6)	0 (0.0)	0 (0.0)	0.200	0.002^*f*^	1.000
Intrathecal *Borrelia*-specific Ab synthesis [no. (%)]	8 (5.1)	7 (100)	1 (12.5)	0 (0.0)	0.001^*f*^	<0.001^*f*^	0.054
Detection of *Borrelia*-specific Abs in blood [no. (%)]	44 (28.2)	7 (100)	6 (75.0)	31 (22.0)	0.467	<0.001^*f*^	0.003^*f*^
Duration of symptoms in days [geometric mean (95% CI)]	60.3 (43.0–83.9)	26.1 (11.0–62.2)	31.8 (11.8–85.6)	64.7 (45.2–93.7)	0.955	0.118	0.182

a CI, confidence interval; CSF, cerebrospinal fluid; Ab, antibody.

b Patients are categorized as definite-Lyme neuroborreliosis (dLNB), possible-LNB (pLNB), or non-LNB cases based on the EFNS criteria (10).

c Three of 7 (42.9%) dLNB patients were selected between August 2013 and June 2016, and 4/7 (57.1%) were from outside this period.

d Six of 8 (75.0%) pLNB patients were selected between August 2013 and June 2016, and 2/8 (25.0%) were from outside this period.

e BH, Benjamini-Hochberg: 1 versus 2, definite- versus possible-LNB patients; 1 versus 3, definite- versus non-LNB patients; and 2 versus 3, possible versus non-LNB patients.

f Significant *P* value after applying the Benjamini-Hochberg procedure (FDR ≤ 5.0%).

### Definite-LNB patients.

Seven (4.5%) of the 156 patients fulfilled the three criteria for definite LNB set by the EFNS (10) (Table 1). Two (28.6%) of them had a positive *Borrelia* species PCR result (Table 1). Six (85.7%) of the seven definite-LNB patients had radiculopathy, of whom two also had cognitive impairment. One patient also had cranial and peripheral neuropathy. The remaining patient had myelitis transversa. All definite-LNB patients were treated with ceftriaxone (2 g/day) intravenously for either 14 (*n* = 4) or 28 (*n* = 3) days, following the Dutch guidelines for LB (52); the treatment was started after the LP was performed (data not shown).

### Possible-LNB patients.

Eight (5.1%) of the 156 patients fulfilled two of the three EFNS criteria for definite LNB (10) and, thus, were classified as possible-LNB patients (Table 1). None of these patients had a positive *Borrelia* species PCR result (Table 1). LNB-specific symptoms among the possible-LNB patients included radiculopathy (*n* = 1; 12.5%), cranial neuropathy (*n* = 6; 75.0%), or peripheral neuropathy (*n* = 1; 12.5%). All of the possible-LNB patients were treated for LNB according to the Dutch guidelines for LB (52); the treatment was started after the LP was performed (data not shown). Six (75.0%) of the possible-LNB patients received ceftriaxone (2 g/day) intravenously for either 14 (*n* = 5) or 28 (*n* = 1) days. One possible-LNB patient had started with intravenous ceftriaxone (2 g/day) but after 5 days switched to oral doxycycline (100 mg twice a day) for 25 days because of an allergic reaction. The remaining possible-LNB patient received oral doxycycline (100 mg twice a day) from the start for 30 days (data not shown).

### Non-LNB patients.

One hundred forty-one (90.4%) of the 156 patients were classified as non-LNB patients, as they did not fulfil at least two of the three EFNS criteria for definite LNB (Table 1) (10). None of them had a positive *Borrelia* species PCR result (Table 1). The five non-LNB patients for whom the CSF cell count was the highest (range, 39 to 821 cells/μl) were diagnosed with neurosyphilis (*n* = 1), Streptococcus meningitis (*n* = 1), tuberculous meningitis (*n* = 1), and viral meningitis (*n* = 2). Other diagnoses that were found at least twice among the non-LNB patients included peripheral neuropathy (*n* = 22), demyelinating conditions (*n* = 17) that included seven cases of multiple sclerosis, radiculopathy (*n* = 9), non-CSF infectious disease (*n* = 8), spinal stenosis (*n* = 7), (transient) facial nerve paralysis (*n* = 6), nonspecific tendon-myogenic pain (*n* = 4), cerebrovascular accident (*n* = 3), microvascular white matter lesions (*n* = 3), headache/migraine (*n* = 3), cancer (*n* = 3), residual complaints after a previous CSF infection (*n* = 3; LNB, neurosyphilis, and viral meningitis), epilepsy (*n* = 2), sleep disorder (*n* = 2), psychogenic disorder (*n* = 2), and arthralgia (*n* = 2). Eighteen non-LNB patients had a diagnosis that was found to be unique in this study population; for 22 non-LNB patients, a diagnosis was never established (data not shown).

### CXCL13 concentrations in the CSF.

**FIG 2:**
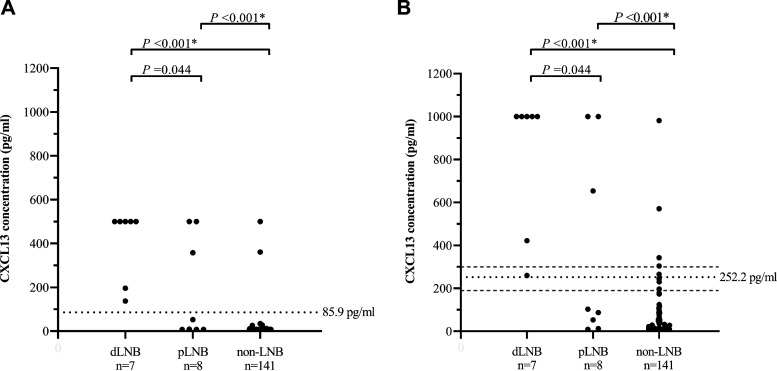
CSF CXCL13 concentrations among seven definite-Lyme neuroborreliosis (dLNB) patients, eight possible-Lyme neuroborreliosis (pLNB) patients, and 141 non-Lyme neuroborreliosis (non-LNB) patients determined by using the CXCL13 ELISA (A) and the *recom*Bead CXCL13 assay (B). The *P* values displayed are raw *P* values that were found to be either significant (FDR ≤ 5.0%; marked with an asterisk) or not (FDR > 5.0%) after applying the Benjamini-Hochberg procedure to account for the multiple comparisons in this study. The (black) dotted horizontal lines in panels A and B are the cutoff values calculated by using ROC curve analysis. The (gray) dashed horizontal lines in panel B are the cutoff values based on the instruction manual of the manufacturer (negative, <190 pg/ml [below lower dashed line]; borderline, 190 to 300 pg/ml [between the dashed lines]; and positive, >300 pg/ml [above upper dashed line]) (40).

Overall, the median CSF CXCL13 concentrations measured by using the *recom*Bead CXCL13 assay were higher than those measured by using the CXCL13 ELISA. Both assays, however, showed similar distributions between the three EFNS groups (10), with the highest CSF CXCL13 levels among the definite-LNB patients (Fig. 2). Using the CXCL13 ELISA, the CSF CXCL13 concentrations among the definite-LNB patients ranged from 138 to 500 pg/ml, with a median CSF CXCL13 concentration of 500 pg/ml (Fig. 2A). This was higher than the median CSF CXCL13 concentration of 30.9 pg/ml (range, 7.81 to 500 pg/ml) among the possible-LNB patients, but not significant (*P* = 0.044; FDR > 5.0%). The median CSF CXCL13 concentration among the non-LNB patients was 7.81 pg/ml (range, 7.81 to 500 pg/ml), and this was significantly lower (*P* < 0.001; FDR ≤ 5.0%) than among both the definite-LNB and the possible-LNB patients (Fig. 2A).

The CSF CXCL13 concentrations among definite-LNB patients measured by using the *recom*Bead CXCL13 assay ranged from 260 to 1,000 pg/ml, with a median of 1,000 pg/ml (Fig. 2B). Among possible-LNB patients, the median CSF CXCL13 concentration was 94.9 pg/ml (range, 9.00 to 1,000 pg/ml), which was lower than the median CSF CXCL13 concentration among definite-LNB patients, although this was not significant (*P* = 0.044; FDR > 5.0%) (Fig. 2B). The median CSF CXCL13 concentration of 9.00 pg/ml (range, 9.00 to 982 pg/ml) among the non-LNB patients was the lowest and was significantly lower (*P* < 0.001; FDR ≤ 5.0%) than among both the definite-LNB and possible-LNB patients (Fig. 2B).

### Diagnostic performance of the CXCL13 assays.

To assess the diagnostic performance of the CXCL13 ELISA and the *recom*Bead CXCL13 assay, ROC curves were created by comparison of the CXCL13 concentrations among cases (i.e., definite-LNB patients) with those among controls (i.e., non-LNB patients). For both assays, the optimal cutoff value was assessed by using the point on the ROC curve for which the distance to the upper left corner, where both sensitivity and specificity are 100%, was shortest. For the CXCL13 ELISA, the optimal cutoff value was determined to be 85.9 pg/ml, with a sensitivity of 100% and a specificity of 98.6%. For the *recom*Bead CXCL13 assay, the optimal cutoff value was determined to be 252.2 pg/ml, with a sensitivity of 100.0% and a specificity of 97.2% (Table 2). This optimal cutoff value fell within the borderline range (190 to 300 pg/ml) recommended by the manufacturer of the *recom*Bead CXCL13 assay (40). The NPV was 100% for both assays, and the PPV was 77.8% for the CXCL13 ELISA and 63.6% for the *recom*Bead CXCL13 assay. No significant difference (*P* = 0.048; FDR > 5.0%) was found between the diagnostic performance of the two assays (Table 2).

**TABLE 2:** Diagnostic performance of the CXCL13 ELISA and the *recom*Bead CXCL13 assay in distinguishing definite-LNB patients from non-LNB patients

Assay	Value (95% CI) for^*a*^:						Raw *P* value for BH comparison
	Threshold (pg/ml)	AUC	% sensitivity	% specificity	PPV (%)	NPV (%)	
CXCL13 ELISA	85.9 (74.7–430.4)	0.993 (0.983–1.000)	100 (100–100)	98.6 (96.5–100)	77.8 (58.3–100)	100 (100–100)	0.048^*b*^
*recom*Bead CXCL13 assay	252.2 (228.1–990.8)	0.993 (0.981–1.000)	100 (100–100)	97.2 (93.6–100)	63.6 (43.8–100)	100 (100–100)	

a *n* = 7 definite-LNB patients and *n* = 141 non-LNB patients. CI, confidence interval; AUC, area under the curve; PPV, positive predictive value; NPV, negative predictive value; BH, Benjamini-Hochberg.

b Nonsignificant *P* value after applying the Benjamini-Hochberg procedure (FDR > 5.0%).

### Interpretation of CXCL13 results.

Twelve (7.7%) of the 156 patients included in this study had a positive CSF CXCL13 result in both assays, which included all (*n* = 7; 100%) definite-LNB patients, three (37.5%) possible-LNB patients, and two (1.4%) non-LNB patients. Five (71.4%) of the seven definite-LNB patients had CSF CXCL13 concentrations above the upper detection limits in both assays. The duration of their symptoms ranged from 21 to 108 days. In contrast, the two definite-LNB patients with shorter durations of symptoms had lower CXCL13 concentrations (both within the detection limits in the two assays). One of them, who had CSF CXCL13 concentrations of 138 pg/ml in the CXCL13 ELISA and 260 pg/ml in the *recom*Bead CXCL13 assay, had had symptoms for 3 days. The other had had symptoms for 14 days and had CSF CXCL13 concentrations of 196 pg/ml in the CXCL13 ELISA and 421 pg/ml in the *recom*Bead assay.

The three possible-LNB patients with positive CSF CXCL13 results in both assays all had pleocytosis. For two of them, the CSF CXCL13 concentrations in both assays exceeded the upper detection limits of the assays. The third possible-LNB patient had CSF CXCL13 concentrations of 358 pg/ml in the CXCL13 ELISA and 654 pg/ml in the *recom*Bead CXCL13 assay. This patient had a shorter duration of symptoms (22 days) than the other two patients (47 and 174 days, respectively).

**FIG 3:**
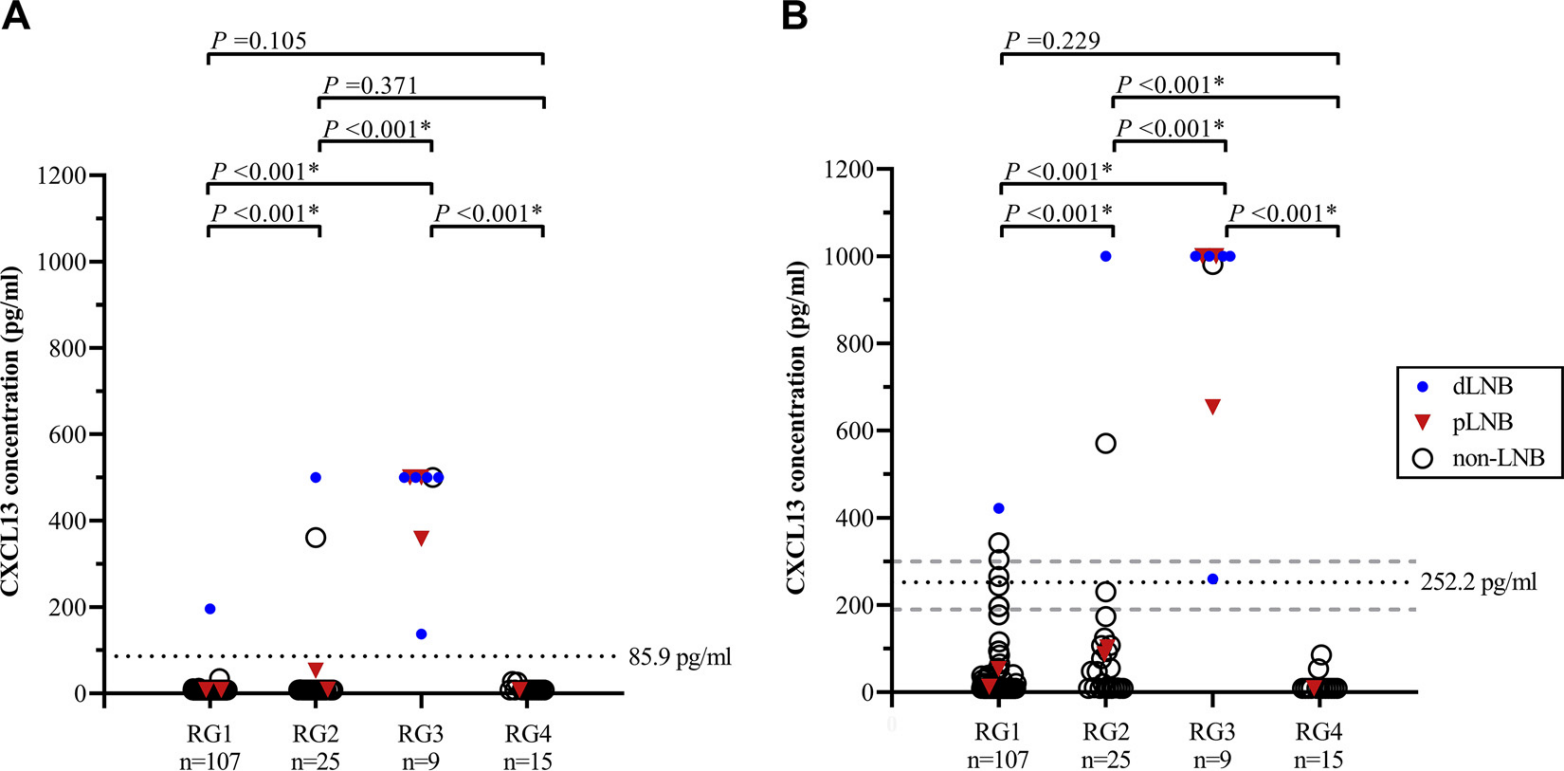
CSF CXCL13 concentrations in the CSF among the 156 patients included in the study, measured by using either the CXCL13 ELISA (A) or the *recom*Bead CXCL13 assay (B), are grouped by Reibergram classification (44). The likelihood of patients having Lyme neuroborreliosis (LNB) as defined by the EFNS (10) is marked as shown in the key in panel B: dLNB, definite-LNB patients; pLNB, possible-LNB patients; and non-LNB, non-LNB patients. The Reibergram classification is based on the combined IgM and IgG Reibergrams (Fig. S1) as shown in Table S2 (RG1, Reibergram group 1; RG2, Reibergram group 2; RG3, Reibergram group 3; and RG4, Reibergram group 4). The *P* values displayed are raw *P* values that were either found significant (FDR ≤ 5.0%; marked with an asterisk) or not (FDR > 5.0%) after applying the Benjamini-Hochberg procedure to account for the multiple comparisons in this study. The (black) dotted horizontal lines in panels A and B are the cutoff values calculated by using ROC curve analyses. The (gray) dashed horizontal lines in panel B are the cutoff values based on the instruction manual of the manufacturer (negative, <190 pg/ml [below lower dashed line]; borderline, 190 to 300 pg/ml [between the dashed lines]; and positive, >300 pg/ml [above upper dashed line)] (40).

The two non-LNB patients who had positive CSF CXCL13 results in both assays were the ones diagnosed with neurosyphilis and tuberculous meningitis. These two patients represented 1.4% of all non-LNB patients. The patient with tuberculous meningitis had CSF CXCL13 concentrations of 361 pg/ml in the CXCL13 ELISA and 571 pg/ml in the *recom*Bead CXCL13 assay. The patient with neurosyphilis had CSF CXCL13 concentrations of 500 pg/ml in the CXCL13 ELISA and 982 pg/ml in the *recom*Bead CXCL13 assay. Three (2.1%) of the 141 non-LNB patients had a positive CSF CXCL13 result in the *recom*Bead CXCL13 assay only (Fig. 2B). These three patients had a normal blood-CSF barrier, no intrathecal total-antibody synthesis (Reibergram area 1) (Fig. 3B), and no pleocytosis. They were diagnosed with (i) a cardiovascular accident and sepsis, (ii) peripheral neuropathy, and (iii) femoral neuropathy.

### CSF CXCL13 concentration versus blood-CSF barrier functionality and intrathecal total-antibody synthesis.

The construction of the Reibergrams (45) provided insight into the functionality of the blood-CSF barrier and the origin of the total IgM and IgG that was detected in the CSF (Fig. S1 in the supplemental material). Patients were subsequently classified in one of four of the five groups based on their position in the Reibergram (Table S2). Analogous to the EFNS groups described above, the CSF CXCL13 concentrations among the four Reibergram groups showed a similar distribution for both CXCL13 assays (Fig. 3).

A total of 107 (68.6%) patients had a normal blood-CSF barrier in the absence of intrathecal total-antibody synthesis and, thus, were classified in Reibergram group 1 (Fig. 3). Only one (0.9%) of them, a definite-LNB patient, had a positive CSF CXCL13 result in both assays. Twenty-five (16.0%) of the 156 patients had a disturbed blood-CSF barrier in the absence of intrathecal total-antibody synthesis and were consequently classified in Reibergram group 2 (Fig. 3). Two (8.0%) of them had elevated CSF CXCL13 levels; one was diagnosed with definite LNB, the other one was the one diagnosed with tuberculous meningitis. The highest CSF CXCL13 levels were found among patients in Reibergram group 3 (Fig. 3). These levels were significantly higher (*P* < 0.001; FDR ≤ 5.0%) than the levels in the other three groups. Nine (5.8%) of the 156 patients were classified in this group, as they had a dysfunctional blood-CSF barrier and intrathecally produced total antibodies. Six of these nine patients had intrathecal total-antibody synthesis of both the IgM and the IgG class (Table S2). Five of them were diagnosed with definite LNB, and the other patient was the one diagnosed with neurosyphilis. The remaining three patients were diagnosed with possible LNB, and all of them had intrathecal total-antibody synthesis of the IgM class only. Fifteen (9.6%) of the 156 patients had a normal blood-CSF barrier and proof of intrathecal total-antibody synthesis; however, none of them had elevated CSF CXCL13 levels in either of the CXCL13 assays (Reibergram group 4) (Fig. 3). None of the patients in the study were classified in Reibergram group 5.

## DISCUSSION

In this retrospective study, we evaluated the diagnostic potential of the chemokine CXCL13 in diagnosing LNB among all consecutive patients who had had an LP in the routine clinical setting of our hospital and who fulfilled the inclusion criteria. Two assays were used to measure the CXCL13 concentrations in CSF, the CXCL13 ELISA and the *recom*Bead CXCL13 assay. Significantly higher CSF CXCL13 levels were found among definite-LNB patients than among non-LNB patients (*P* < 0.001; FDR ≤ 5.0%). Our study results are in accordance with the results published by other research groups (20, 23, 35, 36) and confirm the usefulness of CXCL13 detection in CSF as a marker in the diagnosis of LNB.

Our study suggests that CXCL13 can be of added value for patients classified as possible-LNB patients based on the EFNS guidelines (10). The classification of possible-LNB cases can be a matter of dispute, as this is based on clinical symptoms and either pleocytosis or intrathecal *Borrelia*-specific antibody synthesis. Various studies report higher CSF CXCL13 levels for possible-LNB patients with pleocytosis than with intrathecal *Borrelia*-specific antibody synthesis (14, 35), which we also find in our study. It has been suggested that possible-LNB patients with pleocytosis represent early LNB cases for whom the antibody response still had undetectable levels and that possible-LNB patients with intrathecal *Borrelia*-specific antibody synthesis most likely have had a previous LNB or possibly another disease (14, 35). Long-term persistence of *Borrelia*-specific antibodies in the CSF after successful treatment of LNB has been described (11, 13). In the current study, eight possible-LNB patients were included, of whom three (37.5%) had a positive CSF CXCL13 result in both assays. These three patients had pleocytosis and, following the aforementioned rationale, most likely had an early LNB. Analysis of the IgM and IgG Reibergrams showed that they had a disturbed blood-CSF barrier and intrathecal total-IgM synthesis in the absence of intrathecal total-IgG synthesis, which also supports the diagnosis of an early LNB. In our larger validation study (T. van Gorkom, W. Voet, G. H. J. van Arkel, M. Heron, S. F. T. Thijsen, and K. Kremer, unpublished data), in which we validated five commercial LNB assays, these three patients had intrathecal *Borrelia*-specific antibody synthesis detected by two or more of the LNB assays under investigation. This suggests that the sensitivity of the IDEIA LNB assay used in the current study is lower in the early stages of LNB, as has been reported previously (11, 53). Four (50.0%) of the eight possible-LNB patients with pleocytosis had a negative CSF CXCL13 result in both assays. These four patients had a rather short duration of symptoms (range, 5 to 39 days), and three of them also had relatively low CSF cell counts (range, 6 to 21 cells/μl). A low CSF cell count and a negative CSF CXCL13 result among LNB patients has also been observed by Markowicz et al. (54). The fourth patient for whom the CSF cell count was much higher (i.e., 207 cells/μl) had elevated CSF CXCL13 levels in both assays, but these levels were below the cutoff values of the two assays. Since another diagnosis was never made for these four patients and they responded well to antibiotic treatment, LNB was the most likely diagnosis. The last patient who was classified as a possible-LNB patient, and who had a negative CSF CXCL13 result in both assays, had intrathecal *Borrelia*-specific antibody synthesis and a normal CSF cell count. This patient reported symptoms for more than 6 months and therefore might have had LNB previously. Others have reported similar cases with a long symptom duration and high levels of intrathecally produced *Borrelia*-specific IgG and have speculated that the inflammation might have been resolved by the time of the LP, as shown by normal CSF cell counts and CSF CXCL13 levels, and that this probably reflects the natural course of the disease (14, 55).

In this study, we also found elevated CXCL13 levels in the CSF of patients with other infectious CNS diseases, such as neurosyphilis and tuberculous meningitis, which has been reported before (21, 26, 27, 30). Elevated CSF CXCL13 levels have also been found in patients with HIV, Cryptococcus neoformans meningitis, congenital toxoplasmosis (21, 28), viral encephalitis (32), viral meningitis (e.g., varicella zoster virus, herpes simplex virus, and tick-borne encephalitis virus [25, 28, 54]), multiple sclerosis (19, 28, 33), and CNS lymphoma (26, 34). This underlines that CSF CXCL13 can be a marker for infection and/or inflammation of the CNS and not a specific marker for LNB. This prompted us to investigate the association between the CSF CXCL13 levels and the classification according to Reiber (44) based on the blood-CSF barrier functionality and the presence of intrathecal total-antibody synthesis among LNB patients. Both a dysfunctional blood-CSF barrier and intrathecal total-IgM synthesis are often found among patients with LNB (56–58). In our study, all patients with a dysfunctional blood-CSF barrier and proof of intrathecal total-antibody synthesis (Reibergram area 3 [i.e., group 3]) had positive CSF CXCL13 results in both assays. The majority (8/9; 88.9%) of them either had definite LNB (*n* = 5) or possible LNB (*n* = 3). These results indicate a clear association between elevated CSF CXCL13 levels, a dysfunctional blood-CSF barrier, and intrathecal total-antibody synthesis among LNB patients. The three possible-LNB patients lacked intrathecal *Borrelia*-specific antibody synthesis, and this emphasizes the added value of using Reibergrams in the early diagnosis of LNB (56). Nevertheless, positive CSF CXCL13 results in both assays were also found in a definite-LNB patient who had a normal blood-CSF barrier in the absence of intrathecal total-antibody synthesis (Reibergram area 1 [i.e., group 1]) and in two patients (one diagnosed with definite LNB and the other with tuberculous meningitis) who had a dysfunctional blood-CSF barrier and no proof of intrathecal total-antibody synthesis (Reibergram area 2 [i.e., group 2]).

Although the positivity rates of the CXCL13 assays used in this study gave comparable results among the three EFNS groups, the CXCL13 ELISA resulted in lower CXCL13 levels in the CSF than the *recom*Bead CXCL13 assay. This is underlined by the differences between the cutoff values of the two assays, 85.9 pg/ml for the CXCL13 ELISA and 252.2 pg/ml for the *recom*Bead CXCL13 assay. This difference in absolute CSF CXCL13 levels observed between the two assays could have various explanations. The assays are based on different platforms, and different calculation methods were used to assess the CSF CXCL13 concentrations. In the CXCL13 ELISA, a standard curve is calculated in each run based on seven standards with different concentrations (43). In contrast, the *recom*Bead CXCL13 assay uses a one-point quantification by the inclusion of a calibrator in each run which is compared to the batch-dependent 4-PL coordinates of the standard curve stored in the *recom*Quant evaluation software (40). The use of different capture and/or detection antibodies could also explain the differences found (59). Some of these issues have previously been debated by Henningsson et al. and Markowicz et al., both of whom also reported different absolute amounts of CSF CXCL13 for the two assays (35, 54). Henningsson et al. found lower cutoff values than we did (56 pg/ml for the CXCL13 ELISA and 158 pg/ml for the *recom*Bead CXCL13 assay) (35). Similarly, Markowicz et al. also found a lower cutoff value (131 pg/ml) for the *recom*Bead CXCL13 assay (54). The difference between these two studies and our study mainly consists of the study set-up and, consequently, the groups used for the ROC curve analyses. Differences in sample handling, age, and gender could also explain the differences in the CSF CXCL13 cutoff values between the various studies, as has been suggested by Rupprecht et al. (38). The broad range of cutoff values found in the various studies underline the importance of determining a setting-specific cutoff value, irrespective of the assay, as absolute levels differ between different assays, as well as between identical assays validated by different study groups. Also, more research should be done to assess the cause of these differences in absolute levels of CSF CXCL13. These differences are intriguing and raise the question of whether an international reference standard could be defined.

This study had some limitations. First, for some CSF samples the CXCL13 concentration exceeded the upper measurement limit. These samples were not diluted any further to determine the final CSF CXCL13 concentration. Consequently, and also given the limited number of patients, it was not possible to investigate the correlation between CSF CXCL13 levels and symptom duration. Nevertheless, Henningsson et al. also reported a weak positive correlation between higher CSF CXCL13 and a longer symptom duration (60). In contrast, others found either a negative correlation or no correlation at all (25, 31, 61). Since a positive correlation between CSF CXCL13 levels and symptom duration could be very interesting, as this stresses the role of CSF CXCL13 as an early marker for infection, this should be investigated further in a prospective study. Another limitation was the retrospective design of the study. As a consequence, data could be incomplete, which could have had an effect on the results, e.g., if data on antibiotic treatment are lacking. Antibiotic treatment can abrogate antibody production, as well as CSF CXCL13 production, and thus give rise to false-negative results if given prior to the LP, consequently leading to an underestimation of the sensitivity of the assay under investigation (20, 61, 62). In this study, all definite-LNB and possible-LNB patients were treated for LNB according to the Dutch guidelines for LB (52) which had started after the LP was performed. Even though the electronic patient files of the 156 patients included in this study were analyzed extensively prior to the start of the study, information regarding antibiotic treatment could have been lacking for patients who received antibiotics not related to a visit in our hospital in the weeks preceding the LP. Most patients from whom a CSF sample was taken were excluded in this study due to the absence of a serum sample, insufficient amounts of CSF and/or serum, hemolytic CSF, or because they were treated intravenously with IgG (39, 40). We do not expect this would have led to a bias in our study. The percentage of patients with *Borrelia*-specific serum antibodies among the non-LNB patients (22.0%) (Table 1) exceeded that of the general population in the Netherlands (4 to 8%) (52), which suggests that patients with a history of a tick bite and/or previously detected *Borrelia*-specific serum antibodies are more often subjected to an LP. We believe this represents the clinical practice and not a bias in our study. Another limitation was the unknown number of freeze-thaw steps to which the CSF samples had been subjected (all CSF samples were stored at either −20°C or −80°C); however, the number of freeze-thaw steps was expected to be limited. We therefore do not believe this has negatively influenced the results in this study, as CXCL13 is stable when subjected to repeated freeze-thaw cycles, as shown by Hytonen et al. (for up to a maximum of five freeze-thaw cycles) (25) and by personal experience (for up to four freeze-thaw cycles investigated) (data not shown). Rupprecht et al., however, reported a negative correlation between CSF CXCL13 levels and the number of freeze-thaw cycles but does not mention the number of freeze-thaw cycles that was investigated (38). We did find a negative correlation between CSF CXCL13 levels and storage time (for up to 3 days at +4°C) before freezing at −20°C (data not shown), which was also reported by Rupprecht et al. (38). Prolonged storage at either −20°C or −80°C could also have had an effect on the CSF CXCL13 levels, although studies report differently on this issue (26, 28, 38). Another limitation of this study was the limited numbers of definite-LNB and possible-LNB patients who were diagnosed in our hospital. By choosing a cross-sectional design that comprised almost 3 years (from August 2013 to June 2016), the expected number of LNB patients to be included was 15. This number is based on the annual incidence rate of LNB in the Netherlands in 2010 (i.e., 2.6 per 100,000 inhabitants), the number of general hospitals in the Netherlands (*n* = 84) in that year, and the Dutch population in 2010 (16,600,000) (4, 63, 64). In the current study, however, only nine LNB patients (three with definite and six with possible LNB) fulfilled the inclusion criteria. To reach the expected number of 15 LNB patients for the predefined study period, we added six LNB patients from outside this period. Preferably, the study period would have been extended, but this would have cost much more time and testing, as this would also have resulted in a much larger non-LNB group. Since we do not think the composition of the non-LNB group would be any different, we believe the cross-sectional design of the study still holds. Ideally, the results of this study should be confirmed in a prospective design aiming at inclusion of more LNB patients and controls with other proven non-LNB diseases.

As discussed above, this study was based on a cross-sectional design, which can be considered a strength of this study as discussed by Rupprecht et al. and Leeflang et al. (37, 38). Another strength of this study is the use of a well-defined study population. Due to the nature of our study, possible-LNB patients were included for whom insight into the added value of using CSF CXCL13 as a diagnostic assay is of particular interest, especially for those with clinical symptoms suggestive of LNB and pleocytosis, as was discussed by Rupprecht et al. (38).

In conclusion, this retrospective, cross-sectional study confirms that determining the CSF CXCL13 levels can aid in the diagnosis of LNB. None of the definite-LNB patients had a negative CSF CXCL13 result in both assays. Furthermore, we show the added value of this marker for the group of possible-LNB patients who have pleocytosis without intrathecally produced *Borrelia*-specific antibodies.
