# Supplementary material for: The Usefulness of Two CXCL13 Assays on Cerebrospinal Fluid for the Diagnosis of Lyme Neuroborreliosis: a Retrospective Study in a Routine Clinical Setting
Source: J Clin Microbiol. 2021 Aug 18;59(9):e00255-21. doi: 10.1128/JCM.00255-21 (PMC8373006; doi:10.1128/JCM.00255-21)
Supplement: Supplemental file 1 — Fig. S1. Download JCM.00255-21-s0001.pdf, PDF file, 0.2 MB [file jcm.00255-21-s0001.pdf]

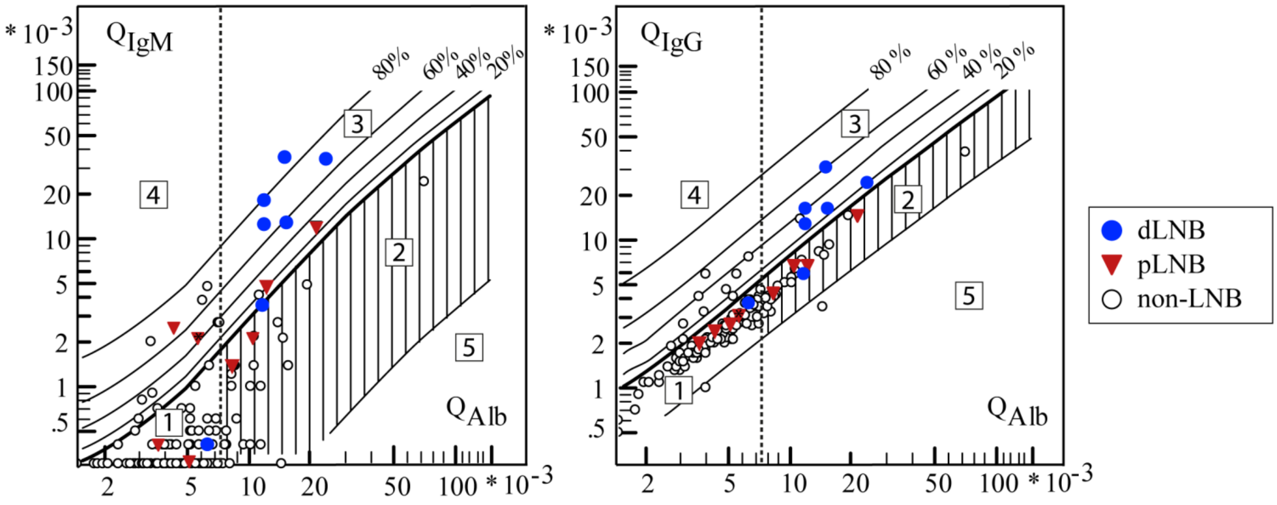

Supplemental Figure S1. CSF/serum quotients for the 156 patients included in the study visualized in CSF/serum quotient diagrams (Reibergrams) for IgM (left) and IgG (right) including the five areas as described by Reiber (1). The black dashed vertical lines represent the average age-dependent reference limit of Q Alb (Q_{lim} Alb) among the 156 patients, which was $7.5*10^{-3}$ (data not shown). The x-axes show the CSF/serum quotients for albumin (Q Alb), and the y-axes show the CSF/serum quotients for total IgM (Q IgM) and total IgG (Q IgG), respectively. Definite Lyme neuroborreliosis (dLNB) patients are indicated in blue dots, possible LNB (pLNB) patients in red triangles, and non-Lyme neuroborreliosis (non-LNB) patients in open circles. One of the eight possible LNB patients was a child for whom the Q_{lim} Alb was $4.7*10^{-3}$ (red triangle with black asterisk) and for whom a dysfunctional blood-CSF barrier was established; however, in the Reibergrams this patient is located to the left of the average Q_{lim} Alb. Two non-LNB patients were located slightly below the lower reference limit of Q IgG, however, they were classified to either the first group (normal blood-CSF barrier and no proof of intrathecal IgG synthesis, or the second group (dysfunctional blood-CSF barrier and no proof of intrathecal IgG synthesis).

Reference

1. Reiber H. 1995. External quality assessment in clinical neurochemistry: survey of analysis for cerebrospinal fluid (CSF) proteins based on CSF/serum quotients. Clin Chem 41:256-63.
