# Supplementary material for: The Usefulness of Two CXCL13 Assays on Cerebrospinal Fluid for the Diagnosis of Lyme Neuroborreliosis: a Retrospective Study in a Routine Clinical Setting
Source: J Clin Microbiol. 2021 Aug 18;59(9):e00255-21. doi: 10.1128/JCM.00255-21 (PMC8373006; doi:10.1128/JCM.00255-21)
Supplement: Supplemental file 2 — Table S2. Download JCM.00255-21-s0002.pdf, PDF file, 0.02 MB [file jcm.00255-21-s0002.pdf]

Supplemental Table S2. Classification of the 156 patients according to their position in the IgM and IgG Reibergram and their overall Reibergram classification.

Area in IgM Reibergram	Area in IgG Reibergram RG1	RG2	RG3	RG4	Total
**RG1**	107[a]			7[b]	114
**RG2**		25[c]			25
**RG3**		3[d]	6[e]		9
**RG4**	7[f]			1[g]	8
**Total**	114	28	6	8	156

All patients were classified according to their position in the IgM and IgG Reibergram as described by Reiber (1), and as is shown in supplemental Figure S1. Following this description, patients with a Q IgM (or Q IgG) located in the first area (Reibergram group 1; RG1) had a normal blood-CSF barrier and absence of intrathecally produced total IgM (or IgG). Patients with a Q IgM (or Q IgG) located in the second area (Reibergram group 2; RG2) had a dysfunctional blood-CSF barrier without proof of intrathecal total IgM (or IgG) synthesis. Patients with a Q IgM (or Q IgG) located in the third area (Reibergram group 3; RG3) had a dysfunctional blood-CSF barrier with proof of intrathecal total IgM (or IgG) synthesis. Patients with a Q IgM (or Q IgG) located in the fourth area (Reibergram group 4; RG4) had a normal blood-CSF barrier and proof of intrathecal total IgM (or IgG) synthesis. Based on the combined IgM and IgG Reibergrams, the overall Reibergram classification was as follows: RG1: 107 patients (white box), RG2: 25 patients (light grey box), RG3, nine patients (dark grey boxes), and RG4: 15 patients (black boxes).

a. One definite, two possible, and 104 non-LNB patients;
b. All non-LNB patients;
c. One definite, two possible, and 22 non-LNB patients;
d. All possible LNB patients;
e. Five definite and one non-LNB patient;
f. One possible and six non-LNB patients;
g. One non-LNB patient.

Reference

1. Reiber H. 1995. External quality assessment in clinical neurochemistry: survey of analysis for cerebrospinal fluid (CSF) proteins based on CSF/serum quotients. Clin Chem 41:256-63.
